# Supplementary material for: Hypermetabolism and Substrate Utilization Rates in Pheochromocytoma and Functional Paraganglioma
Source: Biomedicines. 2022 Aug 16;10(8):1980. doi: 10.3390/biomedicines10081980 (PMC9406117; doi:10.3390/biomedicines10081980)
Supplement: Supplementary file 1 [file biomedicines-10-01980-s001.zip › biomedicines-1770470-supplementary.pdf]

biomedicines MDPI

Table S1. Subjects' basic characteristics according to overproduced catecholamine.

Subjects' characteristics	NOR	ADR	NOR + ADR	*p* (ANOVA)
Subjects, *n* (females)	42 (19)	24 (15)	42 (23)	0.38
Age, y	46 ± 15*	55 ± 12	53 ± 13	0.02
Weight, kg	80 ± 23	78 ± 20	74 ± 16	0.39
Height, cm	173 ± 12*	166 ± 8	170 ± 9	0.03
BMI, kg/m^2	26.4 ± 6.0	27.8 ± 5.4	25.4 ± 5.0	0.25
Waist, cm	90 ± 18	94 ± 18	88 ± 13	0.37
Hip, cm	103 ± 11	106 ± 13	102 ± 9	0.33
WHR	0.87 ± 0.11	0.89 ± 0.11	0.87 ± 0.07	0.70
Body fat percentage, %	31 ± 9	35 ± 10	31 ± 9	0.17
Creatinine, umol/L	74 ± 16	67 ± 15	70 ± 19	0.30
Type 2 DM, *n* (%)	11 (26)	8 (33)	12 (29)	0.83
FBG, mmol/L	5.9 ±.1.8	6.2 ± 1.8	6.0 ± 1.2	0.83
HbA1c, mmol/mol	41 ± 9	45 ± 14	44 ± 9	0.24
Total cholesterol, mmol/L	4.5 ± 0.8 #	4.6 ± 1.1	5.0 ± 1.2	<0.05
Triglycerides, mmol/L	1.3 ± 1.0	1.3 ± 0.6	1.2 ± 0.7	0.93
TSH, uIU/L	1.59 ± 0.94	2.19 ± 1.26	1.88 ± 1.06	<0.05
P_Metanephrine, mmol/L	0.4 (0.2; 0.7) *#	9.6 (3.7; 19.9)	6.4 (2.2; 12.9)	<0.001
Levels above URR	0.8 (0.4; 1.3) *#	18 (7; 37)	24 (4; 24)	<0.001
P_Normetanephrine, mmol/L	11.0 (5.5; 25.0) *	3.7 (1.9; 8.9) ‡	14.5 (4.9; 24.1)	<0.001
Levels above URR	14 (7; 32) *	5 (2; 11) ‡	18 (6; 31)	<0.001
Current Smoker, *n* (%)	11 (26)	5 (21)	14 (22)	0.53
Alpha—blockers, *n* (%)	38 (91)	21 (88)	41 (98)	0.26
Dose of Doxazosine, mg	3 (2; 6)	2 (2; 4)	4 (2; 4)	0.13
Beta-blockers, *n* (%)	16 (38)	11 (46)	18 (43)	0.81
Statin, *n* (%)	10 (24)	6 (25)	13 (31)	0.74

Abbreviations: BMI, body mass index; WHR, waist to hip ratio; DM, diabetes mellitus; FBG, fasting blood glucose; HbA1c, glycated hemoglobin; TSH, thyroid stimulating hormone; P_, plasma; URR, upper reference range; REE, resting energy expenditure. Significant level of statistical significance: * NOR vs. ADR; # NOR vs. NOR + ADR; ‡ ADR vs. NOR + ADR
